# Adapting an integrated neglected tropical disease programme in response to COVID-19

**DOI:** 10.1093/trstmh/traa180

**Published:** 2021-02-11

**Authors:** Amy Clark, Becks Hill, Ron Bannerman, Leah Wohlgemuth, Jennifer Burrill, David Agyemang, Chantelle Genovezos

**Affiliations:** Sightsavers, Chippenham, SN14 6NG, UK; Sightsavers, Chippenham, SN14 6NG, UK; Sightsavers, Chippenham, SN14 6NG, UK; Sightsavers, Chippenham, SN14 6NG, UK; SCI Foundation, London, SE11 5DP, UK; Sightsavers, Accra, PO Box KIA 18190, Ghana; Sightsavers, Chippenham, SN14 6NG, UK

**Keywords:** behaviour change, community engagement, community health workers, coronavirus disease, COVID-19, risk communication

## Abstract

The COVID-19 pandemic hit at a time when the Ascend West and Central Africa programme was nearing the end of its first year of a 3-y programme. This article reflects on key lessons learnt from the rapid adaptation of an integrated neglected tropical disease (NTD) programme to support COVID-19 responses in 11 countries. It shares the experiences of adopting a flexible and directive approach, leveraging the NTD network and relationships, and working in collaboration with multiple ministry departments, commercial sector partners and the UK Foreign Commonwealth Development Office to repurpose over £6 million of budget.

## Background

The Ascend West and Central Africa programme (hereafter called ‘Ascend’, or ‘the programme’) is an ambitious integrated neglected tropical disease (NTD) programme funded by the UK Foreign Commonwealth Development Office (FCDO).^1^ Although led by Sightsavers, it is a consortium comprising the Liverpool School of Tropical Medicine, Mott MacDonald and the SCI Foundation. The consortium works in collaboration with ministries of health alongside other partners, including Accenture Development Partnerships, M&C Saatchi World Services and in-country implementers.

Ascend builds on up to 3 decades of NTD control and elimination efforts across 13 countries and supports beneficiary governments by providing strategic and technical oversight. From April 2019 to March 2022, the programme will support millions of treatments to prevent, treat and accelerate elimination efforts of five NTDs. These diseases include intestinal worms, lymphatic filariasis, river blindness, trachoma and schistosomiasis.

**Figure 1.**
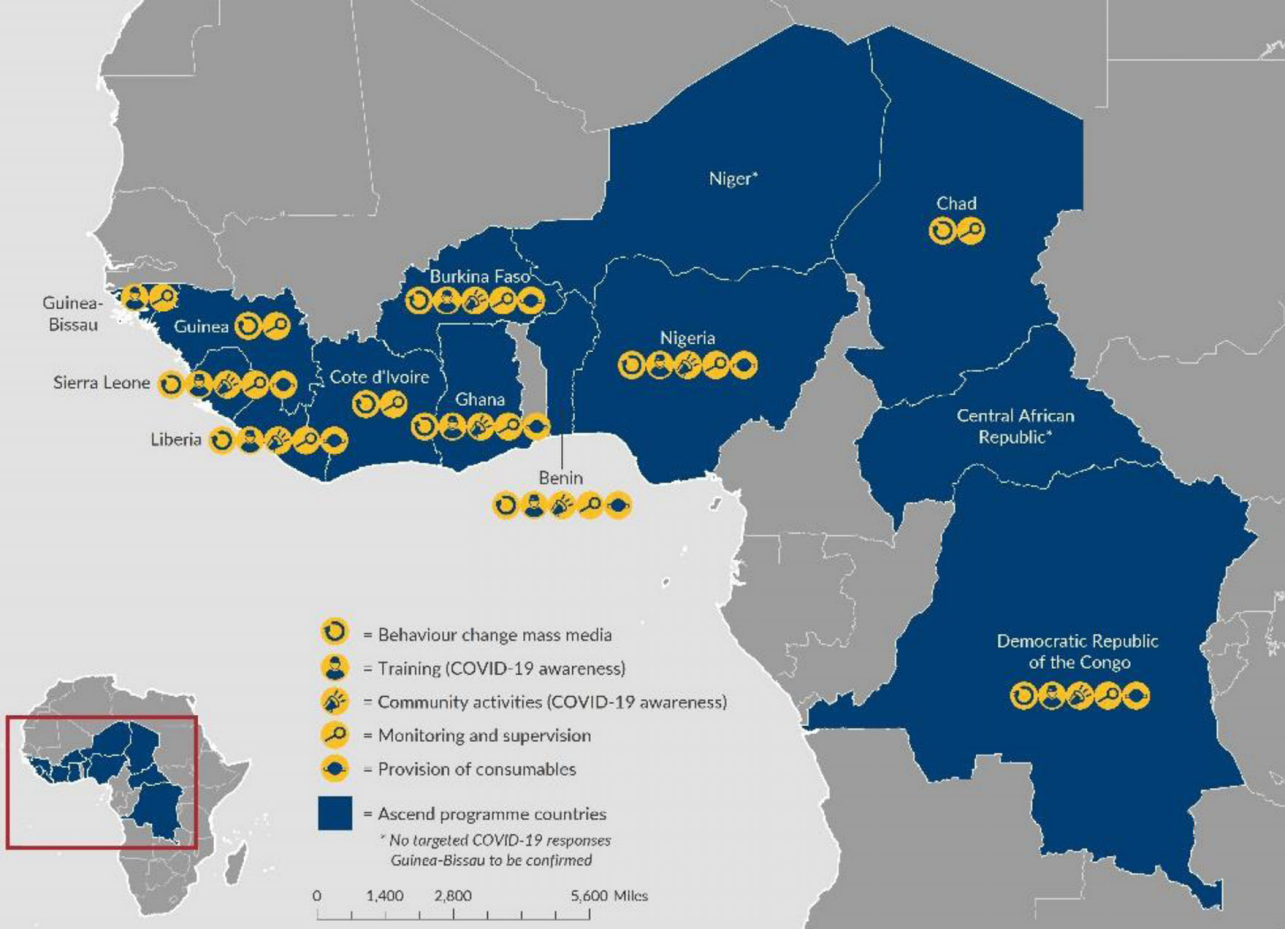
Ascend geographies and COVID-19 activities.

Many of the Ascend geographies (Figure 1) have suffered decades of civil conflict, crippling health systems and face severe shortages of trained health workers. The consortium's approach is to ensure all programme activities are led by national governments and are locally owned. The programme is contributing to strengthening building blocks in national health systems that ensure sustainable delivery of NTD control and elimination activities.

## Introduction

The COVID-19 pandemic hit at a time when the Ascend programme was nearing the end of its successful first year^2^ and was beginning implementation of year 2 activities. On 1 April 2020, the WHO published interim guidance for NTD programmes recommending that ‘community-based surveys, active case-finding, and mass treatment campaigns be postponed until further notice’. Further, the WHO encouraged ‘local health authorities to use existing NTD platforms, surveillance mechanisms and water, sanitation and hygiene/health education opportunities to support implementation of COVID-19-related measures, as appropriate’.^3^

The consortium recognised the opportunity to leverage its expertise, relationships of country teams and the niche NTD programmes offer to public health outreach in an emergency, especially for hard-to-reach communities and people with disabilities.

## Methods

Ascend consortium and country teams worked in consultation with national NTD and other ministry departments to develop 11 country proposals within 2 weeks. These proposals required specific details on the use of funds and impact to beneficiaries (Table 1).

**Table 1.** Contents of the concept note

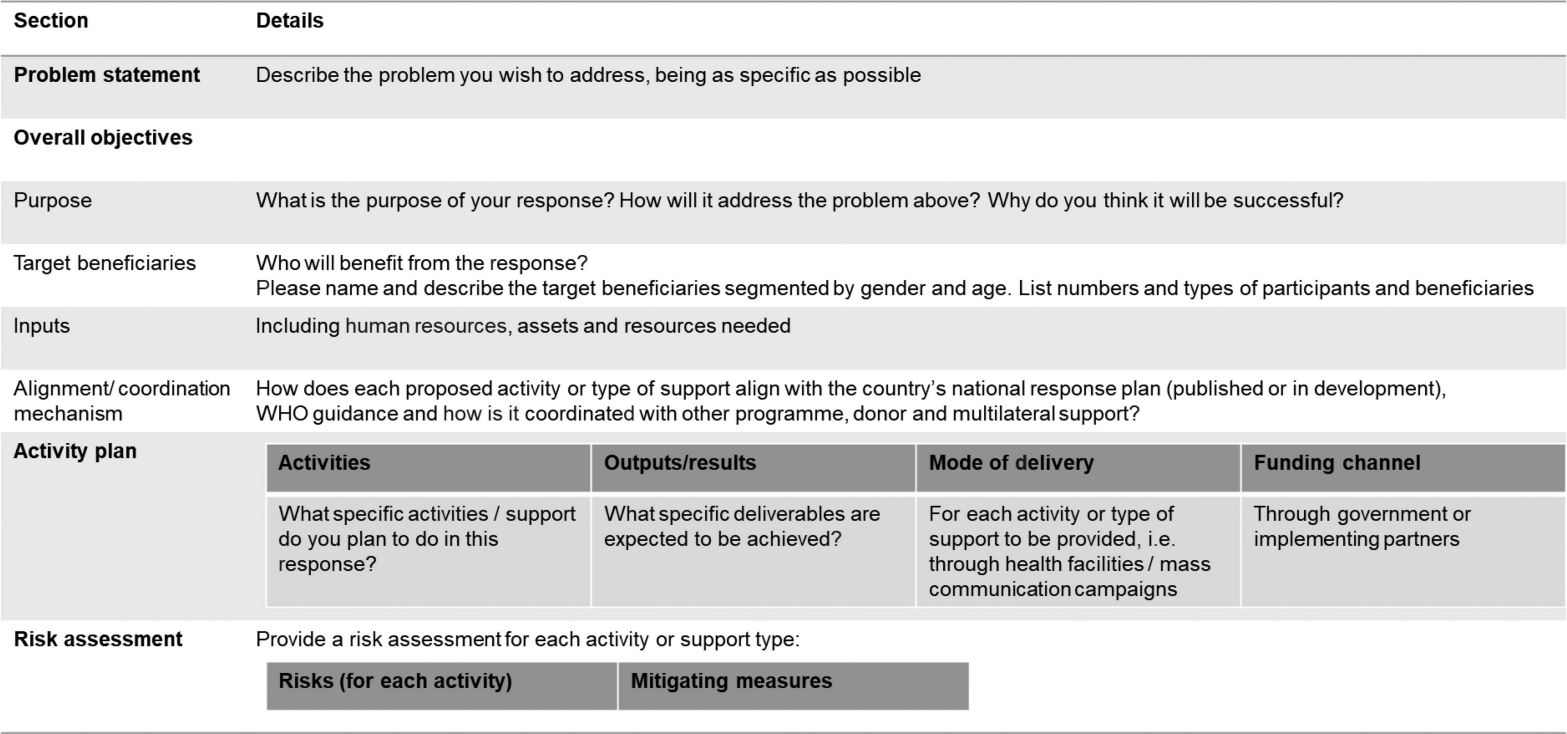

Ascend programme management reviewed the country proposals based on robustness of design, alignment with technical recommendations from the WHO and value for money of the interventions. The review process culminated in final approval to proceed from the FCDO.

## Results

### Rapidly responding to the outbreak of COVID-19

The consortium's deep-rooted relationships with national governments and extensive reach into affected communities meant it was uniquely positioned to support implementation of the nine pillars in the WHO Operational Planning Guidelines to Support Country Preparedness and Response for COVID-19.^4^ As the virus was spreading fast, clear communication and timely information sharing was key. In response, Sightsavers developed a COVID-19 dashboard to consolidate publicly available data from the John Hopkins Corona Virus Resource Centre^5^ to track how the virus was impacting Ascend countries.

It became apparent through ongoing data analysis and continuous engagement with national governments that the consortium could add value to WHO Pillar 2: ‘Risk communication and community engagement’.^6^ While the majority of national governments focused their messaging on handwashing, wearing a mask, physical distancing and combatting misinformation, there was a gap in ensuring these messages were both motivating and accessible to vulnerable groups, particularly people with disabilities, who are often entirely missed out in response measures. Other areas of support identified included repurposing the NTD platforms to support with community contact tracing, addressing misinformation and providing vital sanitation supplies.

Box 1. **Scope of work.**

**11 country proposals**, developed and submitted within **2 weeks** covering up to.

**4 key areas of support:**

■ Contact tracing
■ Training of health workers, teachers, community volunteers
■ Provision of sanitary supplies
■ Mass media campaigns

A rapid collaborative assessment of needs took place at country level, involving M&C Saatchi World Services, who bought technical expertise in delivering cutting-edge behaviour-change communication (BCC) campaigns.^7^ Throughout this process, regular involvement and consultation with the FCDO was vital to assure the prudent use of funds and that activities would not contribute to increased COVID-19 transmission. While this adaptation meant a temporary shift away from NTD control and elimination, it also meant minimising the negative impact of COVID-19 on NTD elimination targets.

### Shifting from business as usual to emergency response

Throughout this process, the consortium adapted ways of working to effectively respond to the emergency while also maintaining business continuity. Leadership and culture were two elements that required rapid, temporary adaptation away from a business as usual comfort zone:

■ Leadership support: experience in humanitarian assistance was identified within the programme management team and these team members were assigned to lead the Ascend COVID-19 response, while operational teams could continue vital health system-strengthening activities to support the continuity and resumption of NTD programmes in the future;
■ Culture shift: providing direct humanitarian assistance required a short-term organisational culture shift, moving away from a methodological to a more directive approach to programme delivery. It demanded quick decision-making and learning what matters. Frequency of communication and interactions with stakeholders significantly increased to meet the demands of the accelerated pace of work, rapid negotiation with governments in each country and uncertainty over the future of NTD programming.

### Building new relationships at country level

The Ascend programme is delivered by NTD departments of ministries of health that the consortium has been working with for many years to provide technical expertise and build capacity. The team rapidly developed relationships with a broader range of ministry departments, communications teams, United Nations bodies, non-governmental organisations and communication networks, all the time working remotely. There was a high risk of duplication of effort and often delays in agreeing country-level support. This was avoided by planning activities in collaboration with the COVID-19 coordination mechanisms in each country, including securing membership at COVID-19 Task Forces, to reduce the risk of undermining national response efforts. In Ghana, to speed up the decision-making process, remote interviews were conducted with ministry directors and WhatsApp groups were established to enhance communication. In Liberia, there were multiple changes to the proposed scope of work as a multitude of stakeholders were supporting the risk communication pillar. A key lesson learnt has been to engage at a ministerial level, where major decisions need to be taken, to ensure alignment with the national COVID-19 effort. In Niger, which was not one of the 11 countries taken forward, the risk of duplication was too high and, despite extensive efforts to define the scope of work, the decision was taken to withdraw Ascend support for any COVID-19-related efforts.

### Working with behaviour-change experts and understanding the country context

Supporting an emergency response demanded new approaches and new partnerships. The Ascend experience demonstrated the value that commercial sector partners can play in COVID-19 health messaging. M&C Saatchi World Services were contracted under Ascend to lead on the BCC component, an integral element of the programme's approach to ensure sustained, lasting impact of NTD control and elimination efforts. As a specialist agency, M&C Saatchi World Service are set up to harness the best of the M&C Saatchi global network of branding and communications expertise, to help create positive change in the world. They had the skills and expertise to facilitate the design of engaging communications, introducing key principles based on proven strategies (Box 2).

Box 2. **Communication principles applied to COVID-19 health messaging supported by Ascend.**

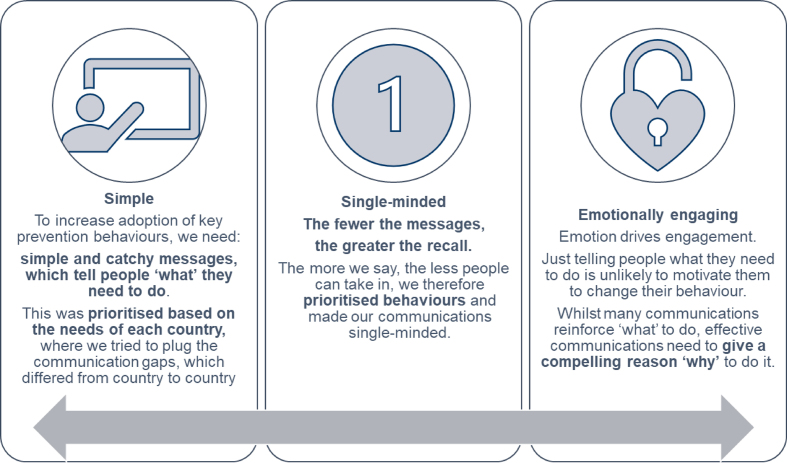

A valuable lesson learnt from the outset was to understand barriers to adopting key prevention behaviours and this required reliance on feedback from ministries of health on the ground and consortium partners. Communication strategies were designed to be emotionally engaging, simple and accessible for people with disabilities, who are often discriminated against in health crisis responses. An apparent challenge was the rapid spread of fake news and conspiracy theories in hindering protective measures against COVID-19. In Nigeria, misinformation on social media was a huge problem that undermined existing prevention advice: many communities did not believe that the virus was a threat or that it even existed.

**Figure 2.**
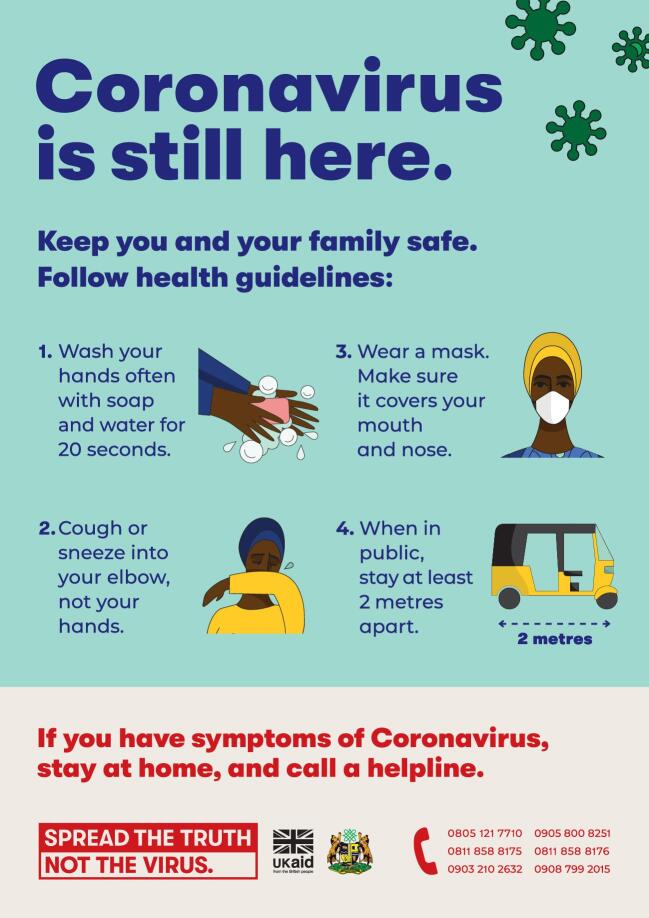
Example of behaviour change communication asset in Nigeria - ‘Spread the truth, not the virus’.

A key lesson learnt was to frame messaging around one piece of key information and to make it memorable and ‘stick’. Often, retaining information in a crisis is diminished, so designing memorable content with simple, catchy slogans can help to ensure this information is remembered. In Nigeria, materials are being developed that focus on the theme of ‘truth’ with the tagline ‘Spread the truth, not the virus’ (Figure 2). However, it is also important to recognise local nuances and to work with governments to assess which messages are appropriate and adapt them as needed to the cultural context.

### Adapting to a new operating environment and building back better

Detailed planning was undertaken to support national NTD activities to ‘build back better’ when it was safe to do so. This required exploring alternative strategies to minimise the risk of transmission of COVID-19, while enhancing efficiency, integration and the consortium's ability to achieve large-scale health impacts. A constant focus on how to ‘build back better’ was maintained to tailor future NTD interventions to this new context. In response, the consortium developed a set of risk assessment and mitigation actions (RAMA) to guide the resumption process. Placing ministries of health in the driving seat of risk management ensures that NTD activities can safely resume, while measures are in place to reduce the transmission risk of COVID-19. The RAMA process and tools were first introduced in Nigeria and Guinea, iterated and improved based on feedback from users and then taken up by other countries.

## Closing remarks

The outbreak of COVID-19 paused NTD activities, yet the FCDO's flexibility supported Ascend to undergo an unprecedented shift to respond to this pandemic. Where and how programme adaptation would be advantageous was supported through data analysis and stakeholder engagement at country level. This ensured Ascend funds and NTD resources were used appropriately and avoided any duplication of efforts with other players in national COVID-19 responses.

Implementation required a rapid change in leadership and culture of the programme, which was introduced by establishing a dedicated task team with a clear mandate. The increased demands on staff are not to be understated and the short-term nature of the response allowed for the intensity of the experience to be withstood. Leveraging existing and establishing new partnerships presents a positive impact on NTD programming, for example, in BCC.

Overall, the experience of adapting an integrated NTD programme to respond to COVID-19 has demonstrated the niche NTD programmes offer to public health outreach in an emergency, especially for hard-to-reach communities and people with disabilities.

Ascend continues to adapt to this new operating environment, all the while supporting governments in the COVID-19 response effort and the resumption of NTD activities to build stronger health systems.

## Data Availability

None.

## References

[1] Sightsavers, Ascend. Fighting disease in West and Central Africa. Available at https://www.sightsavers.org/programmes/ascend/ [accessed 26 November 2020].

[2] Sightsavers. Health programme exceeds several first-year targets, September 2020. Available at https://www.sightsavers.org/news/2020/09/health-programme-exceeds-first-year-targets/ [accessed 26 Nov-ember 2020].

[3] WHO. COVID-19: WHO issues interim guidance for implementation of NTD programmes, 1 April 2020. Available at https://www.who.int/neglected_diseases/news/COVID19-WHO-interim-guidance-implementation-NTD-programmes/en/ [accessed 26 November 2020].

[4] World Health Organization, COVID-19 SPRP Operational Planning Guidelines to Support Country Preparedness and Response, 22 May 2020, https://www.who.int/publications/i/item/draft-operationalplanning-guidance-for-un-country-teams [accessed 5 October 2020].

[5] John Hopkins Corona Virus Resource Center. Available at https://coronavirus.jhu.edu/ [accessed 5 October 2020].

[6] WHO Covid-19 Partners Platform Pillar 2. Available at https://covid19partnersplatform.who.int/pillar/2 [accessed 25 November 2020].

[7] M&C Saatchi. https://mcsaatchi.london/portfolio/change4life/ [access-ed 5 October 2020].

